# Predictors and Consequences of Veterans Affairs Mental Health Provider Burnout: Protocol for a Mixed Methods Study

**DOI:** 10.2196/18345

**Published:** 2020-12-21

**Authors:** Kara Zivin, Jennifer Kononowech, Matthew Boden, Kristen Abraham, Molly Harrod, Rebecca K Sripada, Helen C Kales, Hector A Garcia, Paul Pfeiffer

**Affiliations:** 1 Center for Clinical Management Research Department of Veterans Affairs Ann Arbor, MI United States; 2 Department of Psychiatry University of Michigan Medical School Ann Arbor, MI United States; 3 Program Evaluation and Resource Center and VA Office of Mental Health Operations VA Palo Alto Health Care System Palo Alto, CA United States; 4 Department of Psychology University of Detroit Mercy Detroit, MI United States; 5 Department of Psychiatry and Behavioral Sciences UC Davis Health Sacramento, CA United States; 6 VA Texas Valley Coastal Bend Health Care System Harlingen, TX United States; 7 Department of Psychiatry University of Texas Health Science Center San Antonio, TX United States

**Keywords:** burnout, mental health providers, patient outcomes, provider experience, mental health, veterans

## Abstract

**Background:**

In the Veterans Health Administration (VHA), mental health providers (MHPs) report the second highest level of burnout after primary care physicians. Burnout is defined as increased emotional exhaustion and depersonalization and decreased sense of personal accomplishment at work.

**Objective:**

This study aims to characterize variation in MHP burnout by VHA facility over time, identifying workplace characteristics and practices of high-performing facilities.

**Methods:**

Using both qualitative and quantitative methods, we will evaluate factors that influence MHP burnout and their effects on patient outcomes. We will compile annual survey data on workplace conditions and annual staffing as well as productivity data to assess same and subsequent year provider and patient outcomes reflecting provider and patient experiences. We will conduct interviews with mental health leadership at the facility level and with frontline MHPs sampled based on our quantitative findings. We will present our findings to an expert panel of operational partners, Veterans Affairs clinicians, administrators, policy leaders, and experts in burnout. We will reengage with facilities that participated in the earlier qualitative interviews and will hold focus groups that share results based on our quantitative and qualitative work combined with input from our expert panel. We will broadly disseminate these findings to support the development of actionable policies and approaches to addressing MHP burnout.

**Results:**

This study will assist in developing and testing interventions to improve MHP burnout and employee engagement. Our work will contribute to improvements within VHA and will generate insights for health care delivery, informing efforts to address burnout.

**Conclusions:**

This is the first comprehensive, longitudinal, national, mixed methods study that incorporates different types of MHPs. It will engage MHP leadership and frontline providers in understanding facilitators and barriers to effectively address burnout.

**International Registered Report Identifier (IRRID):**

PRR1-10.2196/18345

## Introduction

### Background

Clinical provider burnout is a key indicator of how well a health care system functions. Health care providers face a large and increasing number of demands to do more work in less time (work compression) to try to achieve the triple aim of improving the patient experience of care, improving the health of populations, and reducing per capita costs of care [1]. Physicians have higher levels of burnout compared to other professionals with advanced degrees and the general US populations of workers [2].

Provider burnout remains a systemic problem associated with reductions in work effort within 24 months [3,4]. Burnout contributes to missed workdays, decreased job satisfaction and engagement, accelerated turnover, premature retirement, and at its most extreme, increased risk of suicide [5-9]. It is estimated that the cost of burnout in a health care system is 3.4%-5.8% of a medical center’s annual operating budget [10]. A meta-analysis found that greater provider burnout was associated with poorer quality health care and reduced patient safety [11].

In the Veterans Health Administration (VHA), mental health providers (MHPs), which include psychiatrists, psychologists, and social workers, report the second highest levels of burnout after primary care physicians [12]. MHPs in the VHA may experience burnout due to factors such as patient violence and suicide, limited resources, changing resources in mental health services, high work demands, inability to effect systemic change, and isolation [13].

MHP burnout gained national attention at the 2017 American Psychiatric Association conference, which featured a crowded town hall session of MHPs sharing their stories of burnout [14]. The VHA recognized this growing area of concern with a number of recent studies focused on burnout at the system and individual levels for physicians and MHPs [15-19]. One study focused on the system level found that the amount of time that VHA psychiatrists spend providing pharmacological intervention increased emotional exhaustion and cynicism scores [16]. At the individual level, VHA tenure appeared strongly associated with burnout, highest for providers with 10-15 years of VHA experience and lowest for those with less than 6 months of Veterans Affairs (VA) service [15].

VHA MHPs face unique challenges compared to other MHPs, increasing their risk of burnout and associated consequences. An external audit found that VHA employees, including MHPs, experience a complex operating environment, including silos, inadequate and often one-way communications, limited access to resources, Congressional inquiries, and ongoing “thrashings” from the press, leading to a lack of empowerment in resolving issues [20]. In addition, the VHA patient population, particularly those with mental disorders, poses more treatment challenges than the private sector patient population, including greater socioeconomic disadvantage; more comorbid medical, psychiatric, and substance use disorders; and poorer self-reported health [21,22].

In light of ample and increasing evidence of negative internal and external pressures leading to MHP burnout, burnout may not soon abate. The VHA recognizes the problem of MHP burnout but could use additional and more nuanced data and guidance regarding potential interventions applicable at both the system and facility levels. Candidate interventions may include implementing team-based care at the system level to reduce MHP isolation [23] and prioritizing hiring clinical and support staff to address resource shortages at individual facilities. VHA MHPs provide care across a wide variation of contexts (eg, telemental health, rural veterans, veterans with complex comorbidities); therefore, our study will examine the range of resources needed both within and across contexts. This study proposes to obtain such information and disseminate findings regarding health system level responses within and outside the VHA.

### Purpose

This article describes a VHA Health Services Research and Development–funded project that will characterize variation in MHP burnout by facility over time and identify workplace characteristics and practices of facilities with low levels of burnout that can be translated for potential implementation at facilities with high levels of burnout. We describe the study objectives and methods and discuss its potential for assisting developing and testing interventions with VHA partners to improve MHP burnout and employee engagement.

## Methods

This study will investigate predictors and consequences of MHP burnout and use these insights for quality improvement within the VHA and elsewhere. We will accomplish 3 aims that focus on assessing both provider and Veteran outcomes to identify existing or new approaches for improving provider working conditions and patient care. Our sequential explanatory mixed methods study aims to understand the factors that contribute to MHP burnout and associated patient outcomes (quantitative), as well as opportunities and challenges that individual facilities experience with trying to address burnout (qualitative) [24]. This will allow us to develop options and recommendations for context-sensitive approaches and interventions for burnout that are acceptable to MHPs and health care system leadership.

### Conceptual Model

**Figure 1:**
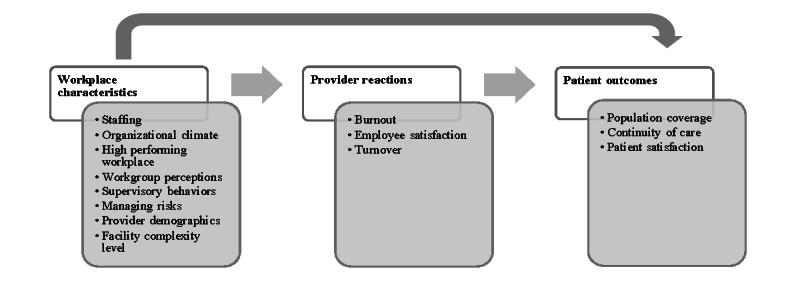
Conceptual model.

The Minimizing Error, Maximizing Outcome (MEMO) Study (Figure 1) guides our project [25]. The MEMO Study examined working conditions in 119 non-VHA primary care clinics across 5 regions and how these conditions affected physician reactions and patient outcomes. The MEMO Study tested 3 hypotheses: (1) unfavorable working conditions would be associated with negative physician reactions such as burnout and (2) poorer patient outcomes, and (3) adverse physician reactions such as burnout to workplace characteristics would be associated with poorer patient outcomes. We will adapt this model to be relevant for mental health care and link VHA survey and administrative data.

### Aim 1: Identifying Predictors of VHA MHP Burnout

We will conduct a quantitative analysis using linked VHA facility-level survey and administrative data to examine predictors and consequences of MHP burnout at VHA facilities (N=141). We will compile annual survey and other data (2014-2018) from 4 VHA sources: All Employee Survey (AES), Mental Health Provider Survey (MHPS), staffing and productivity identified using the Mental Health Outpatient Clinic Method (MHOC), and the SAIL Mental Health Domain (MH-SAIL).

The AES is an annual census of workplace perceptions and satisfaction open to all VHA employees [26]. The annual response rate is 55%-60%. There are no personal identifiers below the workgroup level to ensure anonymity as the survey is focused on organizational improvement needs. There are 3 questions on the AES that are designed to address burnout: (1) I feel burned out from my work (emotional exhaustion), (2) I worry that this job is hardening me emotionally (depersonalization), and (3) I have accomplished many worthwhile things in this job (personal accomplishment). Burnout measures are scored as 0 (never) to 6 (every day).

The MHPS is an annual survey for MHPs designed to assess perceptions about access to and quality of mental health care and overall job satisfaction [27]. The survey is open to all licensed and nonlicensed independent MHPs at the VHA. The annual response rate is ~25%. There is 1 question regarding burnout that is scored from 1 (I enjoy my work. I have no symptoms of burnout) to 5 (I feel completely burned out and often wonder if I can go on).

The MHOC identifies and tracks mental health staffing and productivity in inpatient and outpatient settings across all VHA clinics. We will calculate total mental health annual adjusted productivity (ie, by time spent delivering care) for each provider type (psychiatrists, psychologists, social workers) for each facility. We will primarily use annual staffing and productivity measures but will also conduct sensitivity analyses using quarterly measures.

The MH-SAIL is the mental health domain of the VHA’s quality monitoring system, which is a composite of 3 measures including population coverage, which represents access to care; continuity of care, to assess whether services were provided in a coordinated manner and in the appropriate amount; and experience of care, representing patient-rated treatment experiences and provider-rated assessment of access, quality, coordination of care, and job satisfaction [28]. We will include the provider job satisfaction metrics as part of the provider reactions component of our analyses and the patient experience metrics as part of our patient outcomes analyses. In addition to the 3 composite measures, we will use components of each to create a comprehensive picture of how burnout may be affecting subgroups of patients based on their diagnoses or treatments. These components include percentage of patients with posttraumatic stress disorder receiving psychotherapy for posttraumatic stress disorder and percentage of patients on new antidepressants with 84 days of continuous treatment in order.

Our multilevel analyses clustered at the facility level will incorporate 3 sub-aims: (1) to identify predictors of VA MHP burnout, (2) to examine access and quality of patient care associated with VHA MHP burnout, and (3) to test mediators of the relationship between workplace characteristics and patient outcomes. We present our analytic models in Table 1.

**Table 1:** Sub-aims, models, and data sources for quantitative analyses.

Quantitative aim			Analytical model^a,b^
1a. To identify predictors of VHA^c^ MHP^d^ burnout			ProviderReactions_*ik*_ = WorkplaceCharacteristics_*ik*_ + e_*ik*_
1b. To examine access and quality of patient care associated with VHA MHP burnout			PatientOutcomes_*ik*_ = ProviderReactions_*ik*_ + e_*ik*_
**1c. To test mediators of the relationship between workplace characteristics and patient outcomes**			
	Model 1^e^	PatientOutcomes_*ik*_ = WorkplaceCharacteristics_*ik*_ + e_*ik*_	
	Model 2^f^	PatientOutcomes_*ik*_ = ProviderReactions_*ik*_ + WorkplaceCharacteristics_*ik*_ + e_*ik*_	

^a^We will conduct separate models for each outcome of interest, using both individual predictors and groups of predictors; we will also include (1) year as an additional indicator in the model to account for potential year-to-year variation in findings and (2) facility response rates for All Employee Survey (AES) and Mental Health Provider Survey (MHPS) data.

^b^ProviderReactions is a series of provider reaction measures, including burnout, employee satisfaction, and turnover measures for the _*i*_ year in the _*k*_ facility (AES, MHPS). WorkplaceCharacteristics includes staffing-related covariates for the _*i*_ year in the _*k*_ facility (MHOC); culture-related covariates, including organizational climate, workgroup perceptions, and supervisory behaviors, for the _*i*_ year in the _*k*_ facility (AES, MHPS); and facility-level demographic characteristics for the _*i*_ year in the _*k*_ facility (AES). PatientOutcomes is a series of patient access, continuity of care, and experience measures for the _*i*_ year in the _*k*_ facility (Strategic Analytics for Improvement and Learning [SAIL], MHPS). Finally, e is the error term.

^c^VHA: Veterans Health Administration.

^d^MHP: mental health provider.

^e^Represents the total effect.

^f^Represents the direct effect.

We will use Aim 1 findings to select facilities (N=8), including 4 with high burnout scores and 4 with low burnout scores, for the qualitative phase for our Aim 2 work. We will select sites with varying levels of burnout; if we find several sites with similar levels of burnout, we will include facilities with different sizes, geographic locations, the presence of an academic affiliation, and facility-level patient complexity to increase representativeness of our findings.

For each facility, we will first compute their burnout score using AES survey responses to single items from the Maslach Burnout Inventory including emotional exhaustion (“I feel burned out from my work”) and depersonalization (“I worry that this job is hardening me emotionally”). We will define facility scores by the proportion of providers reporting that either of these 2 statements were true once a week or more frequently. To standardize, we will convert facility burnout scores to a corresponding Z-score by subtracting the overall mean score and dividing by the standard deviation of facility-level burnout scores. Then we will rank facilities into 3 categories based on the magnitude of their Z-score: 0 to 1 for low burnout sites and ≥2 for high burnout sites.

We will select sites primarily based on rankings related to burnout scores; we will not focus on patient outcomes in our site selection. However, if we need to choose between multiple sites with similar burnout scores but differing patient outcomes, we will select sites that allow for more variation in patient outcomes.

### Aim 2: Understanding VHA MHP Leadership and Provider Perspectives Regarding Burnout

Using qualitative methods, we will explore VHA MHP leadership and frontline provider perspectives regarding factors that protect against or exacerbate burnout in facilities with differing levels of burnout. We will conduct semistructured telephone interviews (up to 48) with mental health leadership and frontline MHPs from the 8 facilities identified from Aim 1 findings. Our sample size was chosen based on prior research studies and literature that suggest that, to reach data saturation, between 12 and 50 interviews should be conducted [29,30]. We will work with our operational partners from VA Central Office of Mental Health and Suicide Prevention and the National Center for Organizational Development to identify VHA MHP leadership within the selected facilities to participate in our interviews. We will conduct interviews with mental health leadership and then move to frontline providers. We will ask leadership for a list of frontline MHPs and will randomly select and recruit providers until we identify 5 participants per facility.

Our semistructured interview guides were developed using our conceptual model, MEMO. We will ask leadership questions such as, “How would you describe burnout?” “How do you address provider burnout within your facility?” “What role do you think burnout has in providers’ abilities to care for their patients?” “In the last year, please describe any strategies your hospital has used to address MHP burnout.” We will conclude with, “What do you think your facility needs in order to successfully address burnout among MHPs?” For frontline providers, we will ask them such questions as, “Please describe your experiences with burnout.” “What do you think contributes to provider burnout?” “Has your organization ever provided resources to cope with burnout?” “What suggestions do you have to address burnout among MHPs?”

Our qualitative research team will use a mix of deductive and inductive coding for our analyses. Deductive coding uses previous theories or studies to generate variables or concepts, and our deductive coding will be based on the MEMO model [31]. We will also use inductive coding in which we will carefully examine the data to identify any nuanced themes and categories that are not explained using MEMO. We will create a data codebook that will include definitions and examples of text. We will conduct the qualitative analysis concurrently with the interviews. This will ensure that we can further investigate any themes that are emerging from multiple interviews. We will then use a matrix analysis to organize the data by site and compare sites to determine how each facility experiences burnout, contributing factors, past or existing strategies used to address burnout, and additional beneficial resources identified by the site.

### Aim 3: Identify Strategies to Reduce VHA MHP Burnout

We will identify context-sensitive strategies for facilities to successfully reduce VHA MHP burnout. We will create joint displays (Table 2) by integrating our quantitative (Aim 1) and qualitative (Aim 2) findings to develop an inventory of local strategies to combat burnout as well as identify the facilitators and barriers of MHP burnout. Based on the joint display, the research team will identify potential strategies for both managing burnout and meeting patient needs.

**Table 2:** Sample joint display.

Workplace characteristics (MEMO^a^ model)	Facility	Burnout level^b^	Context (eg, barriers, facilitators)^c^	Strategies tested or considered^c^
Staffing^d^	A	High burnout	Not enough providers to meet patient needs	None
	C	High burnout	Providers feel overworked but get job done	Hire additional support staff
	F	Low burnout	Have right mix of staffing and coordination among providers	Flexible work schedules
	H	Low burnout	Staff do not report being burdened but facility struggles to address required metrics	Request OMHSP^e^ support to meet benchmarks

^a^MEMO: Minimizing Error, Maximizing Outcome.

^b^Based on Aim 1 analyses.

^c^Based on Aim 2 analyses.

^d^Repeat display for all components of MEMO model, including organizational climate, high performing workplace, workgroup perceptions, supervisory behaviors, managing risks, Veterans Affairs initiatives.

^e^OMHSP: Office of Mental Health and Suicide Prevention.

Our exemplar joint displays will be stratified by high and low burnout scores. Using the MEMO model, we will identify barriers and facilitators to addressing burnout in each facility. We will also include any specific strategies that the facility has tested or considered for addressing MHP burnout. Our research team will review the joint display to identify mechanisms that appear most linked with outcomes of interest. For example, our sample joint display suggests that flexible work hours could be a successful strategy for both managing burnout and meeting patient needs.

We will present our findings and understanding of mechanisms associated with MHP burnout to an expert panel including our operational partners, VHA clinicians, administrators, policy leaders, and burnout experts in an online web-based meeting. We will use the Delphi panel protocol, which does not require consensus as each panel member will have a confidential vote [32]. In Round 1, we will send the expert panel a list of potential strategies. Panel members will be asked to rate strategies based on (1) potential impact, (2) acceptability to leadership and providers, and (3) feasibility of implementation and relevance outside of VHA. In Round 2, we will provide an analysis of Round 1 votes and will ask the expert panel members to discuss their rationale. We will then ask the panel members to re-rate the strategies by confidential ballot in terms of the feasibility of implementation based on their experience with or knowledge of the resources available in an average VHA facility.

Our research team will then tabulate the ratings from Round 2 and develop a final set of recommended strategies. The strategies will be chosen based on the panel’s median ratings of impact, acceptability, feasibility, and levels of agreement among panel members. We will not include any strategies that were rated as no or low impact or those that had significant disagreement by the expert panel.

Once the context-sensitive best practices have been identified, we will reengage facilities that participated in Aim 2 and conduct focus groups (up to 16). Prior to the focus groups, we will distribute site-specific integrated findings from the quantitative data, interviews, and expert panel recommendations to participants. The purpose of the focus groups will be to solicit feedback on study findings, which we will then disseminate broadly within and outside VHA.

## Results

This project received notice of intent to fund in October 2018 and received funding to begin the work in December 2019. Institutional review board approval was obtained in July 2019 by the Ann Arbor VA Human Studies Committee. Primary analysis for Aim 1 began in June 2020, recruitment for Aim 2 will begin in March 2021, and Aim 3 work will begin in December 2021.

## Discussion

This study represents the first comprehensive, longitudinal, national, mixed methods analysis to focus on a variety of MHPs. By directly engaging with MHP leadership and frontline providers in the largest integrated health care system in the United States, we will be in a unique position to identify and understand the barriers and facilitators to addressing burnout.

Our work will identify a broad set of recommendations to assist facilities and supervisors address MHP burnout. VHA will be able to use our findings to facilitate future planning and interventions to improve MHP burnout, employee engagement, and patient outcomes. Our work will contribute to broad health care improvements within VHA and beyond and will generate new insights for health care delivery, informing efforts to address burnout in MHPs and other clinical providers. Based on previous research showing the effects of burnout on individual providers and the entire health care system, we believe that our work could improve quality of care and access and reduce costs associated with staff turnover and lost productivity.

We anticipate that we will select, develop, and test the effectiveness of one or more of the selected recommendations and/or study how the recommendations can be successfully implemented. This study will lay important groundwork for a future intervention study or a service directed project, including a randomized program evaluation to study a new practice or policy.

## Abbreviations

AES: All Employee Survey
MEMO: Minimizing Error, Maximizing Outcome
MHOC: Mental Health Outpatient Clinic Method,
MHP: mental health provider
MHPS: Mental Health Provider Survey
MH-SAIL: Mental Health Strategic Analytics for Improvement and Learning
OMHSP: Office of Mental Health and Suicide Prevention
VA: Veterans Affairs
VHA: Veterans Health Administration
