# Correction: Predictors and Consequences of Veterans Affairs Mental Health Provider Burnout: Protocol for a Mixed Methods Study

**DOI:** 10.2196/26934

**Published:** 2021-01-12

**Authors:** Kara Zivin, Jennifer Kononowech, Matthew Boden, Kristen Abraham, Molly Harrod, Rebecca K Sripada, Helen C Kales, Hector A Garcia, Paul Pfeiffer

**Affiliations:** 1 Center for Clinical Management Research Department of Veterans Affairs Ann Arbor, MI United States; 2 Department of Psychiatry University of Michigan Medical School Ann Arbor, MI United States; 3 Program Evaluation and Resource Center and VA Office of Mental Health Operations VA Palo Alto Health Care System Palo Alto, CA United States; 4 Department of Psychology University of Detroit Mercy Detroit, MI United States; 5 Department of Psychiatry and Behavioral Sciences UC Davis Health Sacramento, CA United States; 6 VA Texas Valley Coastal Bend Health Care System Harlingen, TX United States; 7 Department of Psychiatry University of Texas Health Science Center San Antonio, TX United States

In “Predictors and Consequences of Veterans Affairs Mental Health Provider Burnout: Protocol for a Mixed Methods Study” (JMIR Res Protoc 2020;9(12):e18345) the authors noted one error.

The Multimedia Appendix file attached to the originally published article has been removed, as the file contained some private financial information.

The correction will appear in the online version of the paper on the JMIR Publications website on January 12, 2021, together with the publication of this correction notice. Because this was made after submission to PubMed, PubMed Central, and other full-text repositories, the corrected article has also been resubmitted to those repositories.

